# A comparative study on heterogeneous deacetylation of chitin to chitosan under various ultrasound irradiation and characterization

**DOI:** 10.1016/j.ultsonch.2026.107794

**Published:** 2026-02-24

**Authors:** Wenrui Dong, Eugenia Mazzara, Alejandro Sánchez-Baca, Kona Mondal, Mar Villamiel, Ramesh Babu, Da-Wen Sun, Brijesh K. Tiwari

**Affiliations:** a Teagasc Food Research Centre, Ashtown, Dublin 15, Ireland; b Group of Chemistry and Functionality of Carbohydrates and Derivatives, Institute of Food Science Research (CIAL) (CSIC-UAM), C/Nicolás Cabrera, 9, 28049 Madrid, Spain; c The Polymeric Materials and NanoComposite (PMNC) Laboratory, School of Chemistry, CRANN, Trinity College Dublin, Dublin 2, Ireland; d Food Refrigeration and Computerized Food Technology (FRCFT), Agriculture and Food Science Centre, University College Dublin, National University of Ireland, Belfield, Dublin 4, Ireland

**Keywords:** Chitosan, Heterogeneous deacetylation, Ultrasound, Polymer structure

## Abstract

Chitosan, a semi-synthetic polymer derived from the deacetylation of chitin, is a basic polysaccharide widely applied in biomedicine, packaging, and environmental fields. To improve the inefficiency of the conventional heterogeneous deacetylation method relying on prolonged heating under alkaline circumstances, ultrasound-assisted approaches, such as low-frequency probe or ultrasound baths, have been explored as a novel technologies. This study innovatively explores various ultrasound systems as alternative strategies to assist rapid and efficient chitin deacetylation. Different ultrasound set-ups were systematically compared under standardized treatments: low-frequency ultrasound probe (US-L), high-frequency ultrasound plate (US-H), and ultrasound-microwave combination reactor (US-MW) with further structure analysis. Results showed that US-H and US-MW successfully achieved effective deacetylation under the bi-functioning of cavitation and heat effect, while US-L exhibited limited deacetylation performance. Further structural and functional characteristics of the chitosan analogues were confirmed through FTIR, XRD, SEM, TGA-DSC, and HP-SEC, demonstrating a comparable structure of chitosan analogues obtained by US-H and US-MW to conventional deacetylation (CVN), with deacetylation duration significantly reduced from 3 h to 15 min. This work provides foundational insights into potentially scalable and efficient chitosan production, highlighting the potential of US-H and US-MW in sustainable biopolymer manufacturing.

## 1 Introduction

Chitin is the second most abundant natural polysaccharide after cellulose, composed of a highly crystalline poly structure of N-acetyl-d-glucosamine in β-(1 → 4), primarily found in crustacean shells, insect exoskeletons, fungal cell walls, and mollusc materials [1], [2]. Due to its crystalline structure, chitin is highly hydrophobic and insoluble, which limits its application and practical processing [3], [4]. By alkaline deacetylation, chitin is converted to chitosan, forming a basic polysaccharide [5], [6], which exhibits enhanced solubility in acidic media by the protonation of its primary amine groups, thereby enabling broader processing and utilization potential [7], [3]. Chitosan possesses a unique biodegradability, biocompatibility, film-forming ability, antimicrobial and anti-inflammatory properties, making it a successful candidate for mucoadhesive formulations, drug delivery systems, and food packaging films [1], [8], [9], [2].

Conventional chitosan production typically relies on heterogeneous alkaline treatment, where chitin is treated in concentrated NaOH suspensions (30–50% w/w) at elevated temperatures (80–130 °C) for extended durations (1–6 h) [10], [11], [5]. Although enzymatic deacetylation using chitin deacetylase offers an alternative route to chitosan with controlled molecular weight and degree of deacetylation, the industrial application of this method remains limited due to instability, low catalytic efficiency, and high cost [12], [13], [14]. Moreover, microwave assisted deacetylation offering rapid thermal effect and electromagnetic interactions, has emerged as a more efficient and environmentally friendly alternative to produce chitosan, in significantly shorter reaction times (<30 min) and at lower temperatures (50–80 °C), achieving comparable quality to that obtained by traditional methods conducted over 30–120 min [15], [16], [17].

Previous studies on ultrasound assisted deacetylation of chitin have primarily relied on low-frequency ultrasound probes or bath systems [18], [19], [20], [21], [22], [23]. These systems utilize acoustic cavitation to generate microjets and shear forces, which facilitate the disruption of the highly ordered crystalline regions within chitin [24]. This structural breakdown exposes more amorphous regions and reduces diffusion resistance, allowing hydroxide ions to penetrate more deeply into the polymer matrix and react more effectively with acetyl groups [25], [26]. However, US exhibits limited thermal generation capacity, which is not sufficient to meet the demands of endothermic reactions. As a result, effective deacetylation still requires either stringent external temperature control or prolonged non-isothermal treatment durations, typically approaching one h [25], [20], [22]. Furthermore, cavitation-induced mechanical effects may result in partial depolymerization, yielding chitosan of medium to low molecular weight [18], [25]. Different from mechanical cavitation dominating at low ultrasound frequencies (<100 kHz), high-frequency ultrasound (>100 kHz) tends to produce weaker cavitation but results in sono-chemical effects, by free radical-induced bond cleavage, playing a more important role in polymer transformations [27], [28], [29]. Previous work also successfully demonstrated promising protein removal without chemical treatment by high-frequency ultrasound [30], but its effectiveness in deacetylation remains underexplored. Besides, the combination of ultrasound and microwave technologies has been previously reported to synergistically enhance both thermal and non-thermal effects, successfully applied in polysaccharides processing [31], [32], [33], which can be considered as a promising route to realize a feasible deacetylation.

The novelty of this study lies in the comparative evaluation of different ultrasound modalities for heterogeneous deacetylation of chitin. In contrast to previous studies focusing solely on low-frequency ultrasound probe and bath, this work systematically investigated the feasibility of achieving high-degree chitin deacetylation within a short treatment time (15 min) under standardized conditions using three different systems: low-frequency ultrasound probe (US-L), high-frequency ultrasound plate (US-H), and ultrasound-microwave combination reactor (US-MW). This study provides a machine-level comparative analysis of process efficiency to advance understanding of how different energy delivery modes influence deacetylation efficiency and structural outcomes, thereby offering practical insights for industrial optimization of chitosan production. Our findings are expected to provide new time-saving and potentially scalable deacetylation strategies for future technical parameter and chemical consumption optimization in chitosan manufacturing.

## 2 Methodology

### 2.1 Materials and chemicals

Standard practical-grade chitin from shrimp shells (CT, CAS number 1398–61-4, product number C7170), practical-grade chitosan from shrimp shells (CS, CAS number 9012–76-4, product number 417963), sodium hydroxide (≥98% purity), ammonium acetate (≥99% purity), and acetic acid (≥99.7% purity) were purchased from Merck KGaA, Darmstadt, Germany. All solutions were prepared by using Milli Q water (DDW, 18.2 MΩ/cm, Barnstead Nanopure ultrapure water system, Thermo Scientific, Marietta, OH, USA).

### 2.2 Chitin deacetylation process to chitosan

**Fig. 1:**
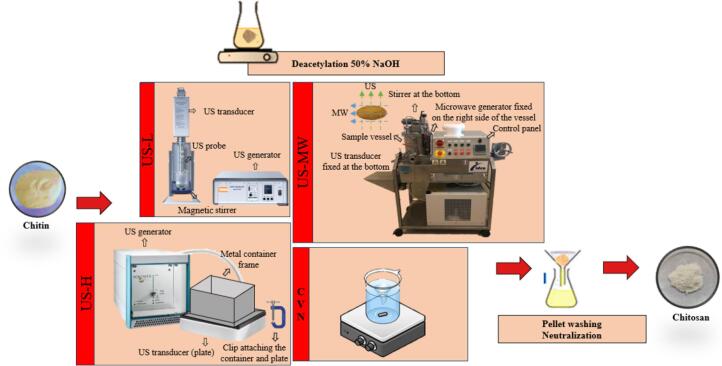
Chitin deacetylation to chitosan by conventional extraction (CVN), low-frequency ultrasound probe (US-L), high-frequency ultrasound plate (US-H), and ultrasound-microwave combination reactor (US-MW). US is ultrasound; MW is microwave.

Deacetylation process of chitin to chitosan was shown as Fig. 1. The investigation was based on the moderate protocol in previous study with modifications [5], using consistently 10 g commercially available chitin powder (Merck KGaA, Darmstadt, Germany), dispersed in fixed 1000 g 50% w/v NaOH (1:100 w/w) in all treatments to avoid the influence by chemical deacetylation conditions. This relatively high liquid load was applied to satisfy the minimum working volume required by the reactors and to ensure comparability among equipment configurations. As a reference of effective deacetylation, the conventional conversion (CVN) was carried out for 3 h at 95 ± 5 °C under nominal 600 rpm magnetic stirring with temperature control. Different ultrasound equipment in chitin deacetylation, a low-frequency ultrasound probe (US-L), a high-frequency ultrasound plate (US-H), and an ultrasound-microwave combination reactor (US-MW) were employed as below:

(i) US-L: A probe-type ultrasound (UIP 1000 hdT, Hielscher Ultrasound technology, Teltow, Germany), adapted with a 40 mm stainless steel probe, generating 20 kHz frequency, 0–1000 W adjustable power ultrasound was used. To keep the substrate homogeneous, magnetic stirring at nominal 600 rpm was continuously applied during the process.
(ii) US-H: A plate-type ultrasound (SONOSYS Ultraschallsysteme GmbH, Germany) comprising a 4-inch plate transducer, generating 400 kHz frequency, 0–500 W adjustable power ultrasound was used. A stainless-steel frame (0.5–5 L) containing the sample was fixed onto the plate with metal clips.
(iii) US-MW: A ultrasound and microwave combination reactor (E200, Idco SAS, 148 Marseille, France) was used, consisting of a cylindrical sample vessel (volume: 0.5–5 L) with a rotary stirrer, and an ultrasound transducer generating 25 kHz and adjustable power 0–200 W ultrasound at its bottom wall, as well as a microwave generator horizontally connected to the vessel wall generating microwave 2 kW / 2450 MHz with adjustable power 300–1950 W. A microwave leak detector was employed consistently during running under safety consideration.

**Fig. 2:**
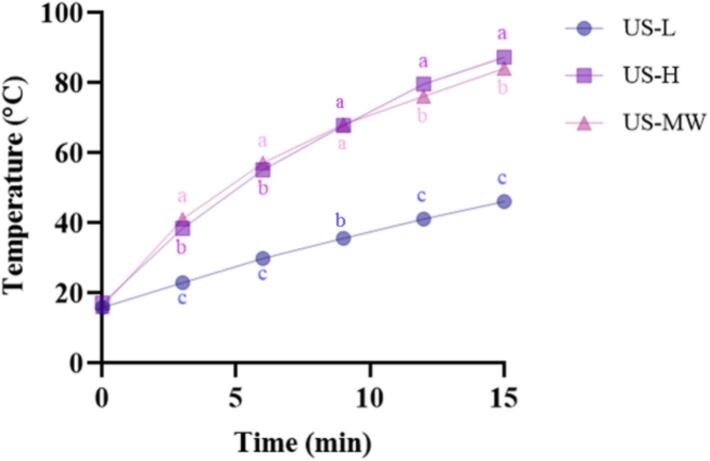
Temperature dynamics for low-frequency ultrasound probe (US-L), high-frequency ultrasound plate (US-H), and ultrasound-microwave combination reactor (US-MW) during chitin deacetylation.

The processing conditions and equipment information employed are summarized as Table 1. To investigate an effective, practical, and application-oriented ultrasound-assisted deacetylation setup, the different ultrasound systems were applied under each maximum power conditions. Since temperature is an active mechanistic driver in alkaline deacetylation, non-isothermal operation without external cooling applied was intentionally maintained to reflect the practical performance of these reactor configurations under process-relevant conditions. A treatment duration of 15 min was applied according to preliminary trials, which ensured sufficient acoustic and thermal exposure for effective deacetylation while minimizing degradation. The temperature increase was recorded every 3 min by a thermometer for CVN, US-L, and US-H, and US-MW temperature values were displayed on the control panel, shown as Fig. 2. All the extraction methods were conducted in triplicate, and the resulting mixtures were continuously washed with Milli-Q water and filtered with a four-layer cloth until the permeate water reached neutral pH. All the retentates were kept in the 50 °C oven-drier until constant weight, and named as CS-C (chitosan analogue obtained by CVN), CS-L (chitosan analogue obtained by US-L), CS-H (chitosan analogue obtained by US-H) and CS-USMW (chitosan analogue obtained by US-MW) respectively. The final recovery quantity was recorded to calculate the recovery yield as formular below.

(1) $$Recovery\;yield\left(\%\right)=\frac{m_{s}}{m_{t}}\times 100$$

where m_s_ is the quantity of dry chitosan obtained after deacetylation, and the m_t_ refers to the commercial chitin used for deacetylation processing.

**Table 1:** Experimental Configurations and Processing Conditions for Conventional Extraction (CVN), Low-Frequency Ultrasound Probe (US-L), High-Frequency Ultrasound Plate (US-H) and Ultrasound-Microwave Combination Reactor (US-MW).

**Name**	**Chitin**	**Duration**	**Solvent**	**Rotation**	**Temperature**	**Ultrasound frequency**	**Ultrasound power**	**Microwave power**	**Maximum capacity**
CVN	10 g	3 h	NaOH 50% (1:100 w/w)	Nominal 600 rpm	95 ± 5 °C	−	−	−	−
US-L	10 g	15 min	NaOH 50% (1:100 w/w)	Nominal 600 rpm	Fig. 2	20 kHz	1000 W	−	5 L, and adaptable into a flow cell under increasing substrate
US-H	10 g	15 min	NaOH 50% (1:100 w/w)	−	Fig. 2	400 kHz	500 W	−	5 L in the used stainless-stell frame, adaptable to 10 L in a metal frame
US-MW	10 g	15 min	NaOH 50% (1:100 w/w)	Nominal 24 rpm	Fig. 2	20 kHz	200 W	1460 W	6 L

### 2.3 Fourier Transform Infrared Spectroscopy (FTIR)

For the characterization of extracted chitosan samples, compared to the commercially available chitin and chitosan powder (Merck KGaA, Darmstadt, Germany), a Spectrum Two Fourier Transform Infrared (FTIR) Spectrometer was used in Attenuated Total Reflection mode (iD7 ATR-Diamond), under the spectral range of 4000–400 cm^−1^, with a resolution of 4 cm^−1^ and an accumulation of 64 scans.

The Degree of Deacetylation (DD) and Degree of Acetylation (DA) were determined from FTIR spectra based on Brugnerotto et al. [34] and El Knidri et al. [35] methods. Specifically, peaks areas at 1320 and 1420 cm^−1^ wavelength were integrated using the specific baseline ranges recommended by Brugnerotto et al. [34], and adapted to the corresponding calculation formulas as below.

(2) $$\mathrm{DA}\left(\%\right)=\left(\frac{Area1320}{Area1420}-0.3822\right)/0.0313$$

(3) *DD* (%) = *100 – DA (%)*

### 2.4 Size exclusion chromatography (HP-SEC)

According to Fiamingo et al. [20] methods with some modifications, the different chitosan analogues were dissolved in 1% acetic acid (1 mg/mL) at 40 °C under 600 rpm shaking overnight, and the solutions were filtered through a 0.45 μm cellulose acetate membrane (Millipore) into vials. All samples in vials were then injected (*v* = 100  μL) into a TSK gel Size Exclusion (PW-type) HPLC column, phase G2500PWxl and G5000PWxl, L x I.D. 30 cm x 7.8 mm, 10 µm particle size by Sigma-Aldrich. High-performance size exclusion chromatography coupled with evaporative light scattering detection (HPSEC-ELSD) was carried out by a LC chromatograph Agilent Technologies 1220 Infinity and a detector ELSD 1260 Infinity (Agilent Technologies, Boeblingen, Germany). The eluent was prepared using 0.075 mol·L^−1^ ammonium acetate / 0.10 mol·L^−1^ acetic acid buffer (pH = 4.5) with sonication for 30 min, and loaded with a constant 0.4 m l·min^−1^ flow rate into the HPSEC-ELSD system. The molecular weights of the different samples were obtained from the SEC profiles by extrapolation in a calibration curve using different known molecular weight pullulan standards ranging from 0.342 to 894 kDa (sourced from Fluka Analytical).

### 2.5 Particle size determination

According to David et al. [36], a Mastersizer 3000 (Aero S Unit, Malvern Instruments Ltd.) working by static light scattering was employed to determine the particle size. Chitosan samples were dispersed in water at a concentration of 0.1% (w/v), and vortex-mixed to form a stable suspension. The refractive and absorption indexes were 1.73 and 0.01, respectively. The General-Purpose model was used, and the obscuration range was 0.10–50%. The samples were stirred at 500 rpm and equilibrated for 90 s inside the instrument before data collection, with the in-built ultrasound dispersion consistently on to prevent the aggregation of suspended particles. Three measurements were performed for all the samples to obtain the particle size distribution.

### 2.6 X-ray diffraction (XRD)

The crystallinity of chitosan samples was analysed using a Bruker D8 Advance X-Ray Diffractogram (XRD) system, equipped with Cu Kα radiation (λ = 1.5418 Å) and operated at 40 kV and 40 mA. XRD patterns were recorded across a 2θ angular range from 5° to 70°, with a step size of 0.0205° per second. The crystallinity percentage (% crystallinity), indicating the crystallinity content of the samples, was determined using the deconvolution method with GRAMS peak fitting software. Gaussian functions were applied to fit the individual peaks corresponding to the XRD patterns of each sample, allowing for the calculation of the area under the various crystalline and amorphous bands. The % crystallinity of all samples, including commercially available chitosan, was determined by dividing the sum of the total area under the crystalline peaks (Ac) by the sum of the total area of the deconvoluted range, which includes both crystalline (Ac) and broad amorphous bands (Aam), as shown in the equation below:

(4) $$\mathrm{Crystallinity}\left(\%\right)=\frac{A_{c}}{A_{c}+A_{\mathrm{am}}}\times 100$$

### 2.7 Thermogravimetric analysis-differential scanning calorimetry (TGA-DSC)

The thermal properties of the chitosan samples, including the commercial polymer, were assessed by simultaneous Thermogravimetric Analysis (TGA) and Differential Scanning Calorimetry (DSC). For this purpose, a TGA/DSC 3 + STARe equipment (METTLER TOLEDO, Ohio, United States) was employed. Chitosan aliquots of 10–15 mg were weighed, sealed in aluminium pans, and heated within the system at a rate of 10 ^◦^C/min and in the temperature range of 10–600 ^◦^C. The analysis was performed at a flow rate of 20 mL/min in an inert N_2_ atmosphere.

### 2.8 Scanning electron microscopy (SEM)

The surface morphology of extracted chitosan powders was characterized using field emission scanning electron microscopy (FESEM, Gemini, Zeiss ULTRA Plus) with an accelerating voltage range of 0.1–30 kV. For imaging, the accelerating voltage was maintained at 3 kV. To ensure conductivity, the samples were coated with a thin layer of gold/palladium alloy using a sputter coater (Cressington 208 Sputter Coater) operated at a current of 40 mA under a vacuum of 0.1 mbar for 40 s.

### 2.9 Statistical analysis

SPSS (Version 29, USA) was used for data analysis. The significance of the analysis was determined by the one-way analysis of variance (ANOVA) and Duncan's multiple tests (P < 0.05). The level of significance (P) for all hypothesis tests was 0.05. All the experiments were repeated 3 times, and the data were expressed as means ± standard deviation.

## 3 Results and discussion

### 3.1 Fourier Transform Infrared Spectroscopy (FTIR) characterization

**Fig. 3:**
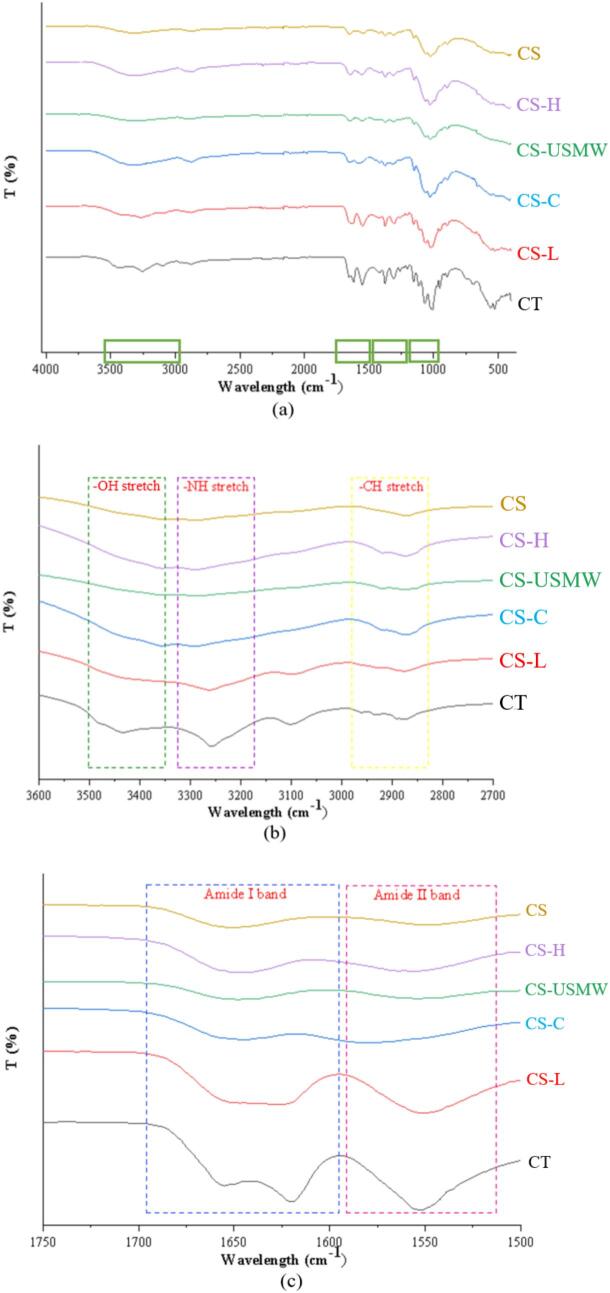

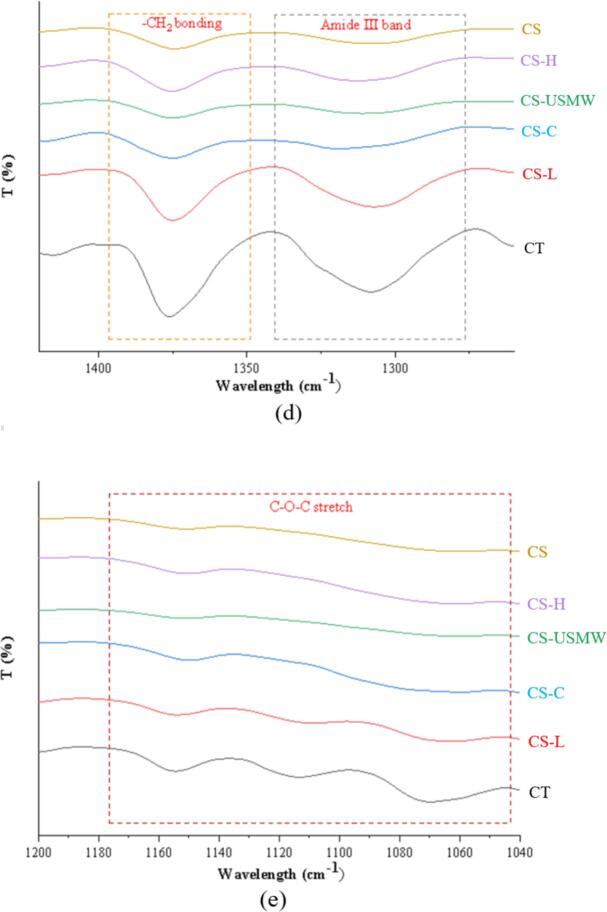
FTIR spectra of commercial chitin (CT), commercial chitosan (CS), and chitosan analogue extractions by conventional extraction (CS-C), low-frequency ultrasound probe (CS-L), high-frequency ultrasound plate (CS-H), and ultrasound-microwave combination reactor (CS-USMW). (a) is full FTIR spectra in the region of 4000 – 400 cm^−1^; (b) is expanded view for –OH and –NH stretches in region of 3600–2700 cm^−1^; (c) is expanded view for Amide I and Amide Ⅱ band in region of 1750–1500 cm^−1^; (d) is expanded view for –CH_2_ bonding and Amide Ⅲ band in region of 1400–1200 cm^−1^; (e) is expanded view for C-O-C stretch in region of 1200–1000 cm^−1.^

The functional groups of chitin and chitosan exhibit distinct absorption patterns in FTIR spectra, reflecting structural modification arising from the deacetylation process. The accompanying Fig. 3 presents the FTIR spectra of commercial chitin (CT), commercial chitosan (CS), and all chitosan analogues. Overall, the spectra of CS-H, CS-USMW, and CS-C closely resemble that of CS, while the CS-L absorption curve aligns with CT, indicating only partial deacetylation in CS-L.

In the regions of 3432–3434 cm^−1^ and 3250–3300 cm^−1^ wavelength, CT exhibited stretching vibration bands corresponding to –OH and –NH groups, respectively, which aligns with previous reports describing chitin –OH and –NH stretching vibrations appearing as two separate bands [37], [38]. In contrast, chitosan displays a broad absorption band where –OH and –NH vibrations overlap, and it has also been recorded as –OH stretching vibrations in some studies [39], [40]. It can be observed that CS, CS-H, CS-USMW, and CS-C spectra converge around 3290 cm^−1^, while CS-L retains more of the dual-peak structure characteristic of CT, providing spectral evidence consistent with its low DD.​ Besides, multiple absorption peaks are observed in the 2870–2920 cm^−1^ range, corresponding to symmetric and asymmetric stretching vibrations of ring –CH, –CH_2_OH, and –CH_3_ groups as reported [41], [39]. Moreover, the amide I band (C=O stretching vibration) serves as a crucial fingerprint region. Chitin typically exhibits a doublet structure around 1620 cm^−1^ and 1655 cm^−1^, whereas chitosan presents a single peak [42], [35], [37], as displayed in CS, CS-H, CS-USMW, and CS-C near 1650 cm^−1^. CS-L shows a less distinct doublet structure with a tendency toward merging, further supporting partial and incomplete deacetylation.​ The amide II band (N-H bending vibration) is another distinguishing fingerprint region for chitosan and chitin. In CS, CS-H, CS-USMW, and CS-C, this band diminishes sharply, while in CS-L and CT, it remains prominent, indicating residual acetyl groups in the polymers, again validating the presence of residual acetyl groups and supporting the analytically determined low DD for CS-L [43], [42]. Additionally, compared to CT and CS-L, the intensities of CH_2_ bending, amide III band, and C-O-C stretching vibration peaks in CS-H, CS-USMW, and CS-C are significantly reduced. This reduction is attributed to degradation, dehydration, deacetylation, and depolymerization reactions of the pyranose ring during deacetylation treatment [40]. Notably, CS-USMW exhibits a smoothing curve with loss of certain key spectral bands. In contrast, CS-H and CS-C display stronger expressions of various functional groups compared to CS, suggesting better preservation of chitosan's molecular structure integrity.

### 3.2 Deacetylation and recovery performance under multiple ultrasound equipment

**Fig. 4:**
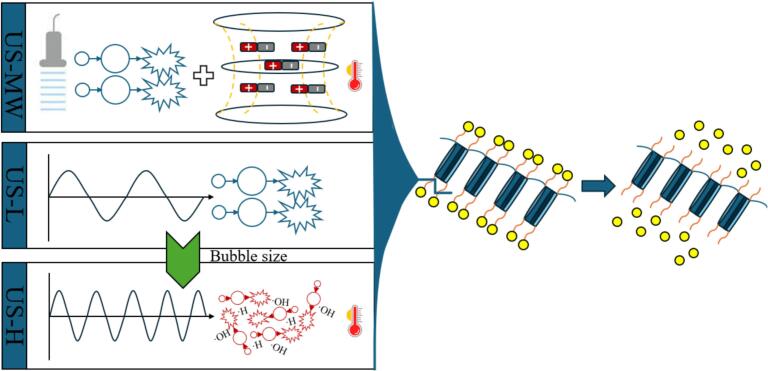
Conceptual mechanistic of deacetylation pathways under low-frequency ultrasound probe (US-L), high-frequency ultrasound plate (US-H), and ultrasound-microwave combination reactor (US-MW).

Table 2 illustrates the deacetylation degree (DD) of chitosan under various treatment methods, and the Fig. 4 interprets the dominant mechanism pathways for each treatment system providing a framework of observed deacetylation and yield performance. Compared with CS-C, CS-H and CS-USMW achieving feasible deacetylation, CS-L only reached 24.80 ± 7.94% DD, retaining most of the chitin structure. This limitation can be attributed to several factors, and the thermal effects generated under non-isothermal ultrasound treatments are likely the primary driver. The system temperature was recorded as shown in Fig. 2, where the temperature of US-H and US-MW reached around 80 ℃, which is noticeably higher than that of US-L (less than 50 ℃). Temperature is one of the most critical parameters in the deacetylation reaction, as it accelerates reactions, promotes hydrolysis, weakens acetyl group bonds, and increases DD [35], [5], [22]. Previous studies have demonstrated that low-frequency ultrasound facilitates mechanical disruption through cavitation, peeling off the layered structure of chitin crystals, exposing more acetyl groups, and increasing hydroxide ion penetration into solid particles [18], [21], of which illustration is shown as Fig. 4. Different from this, it is possible that the cavitation effect of US-L in our case was insufficient to offset heat deficiency within a 15 min reaction, consistent with previous findings that ultrasound-assisted deacetylation at 40–70 °C for 15 min provides below 40% DD, requiring at least 1 h for feasible deacetylation [22]. The CVN produced a medium-to-high DD (77.97 ± 1.34%), comparable to CS, and slightly higher than CS-USMW (73.25 ± 3.84%) without being significantly different (p > 0.05). By combining low-frequency ultrasound cavitation and rapid microwave heating, US-MW greatly reduced deacetylation time from 3 h to just 15 min. Besides, microwave dipolar rotation and induced ionic conduction potentially accelerate local reaction rates and promote heterogeneous deacetylation [44], shown as Fig. 4. With the same processing time, US-H also achieved effective deacetylation (DD 64.65 ± 0.51%), slightly lower than CS-C (p < 0.05) but not significantly different from CS-USMW (p > 0.05). Although US-H has similar thermal effects to US-MW, the mechanical destructive power by numerous smaller cavitation bubbles bursting under high-frequency is milder compared to the explosions of larger bubbles produced under low-frequency ultrasound, which was graphically explained as Fig. 4, reducing the likelihood of fragmentation, penetration, and polymer chains breakage [45], [46]. As the frequency increases over 100 kHz, the ultrasound sono-cavitation is gradually replaced by sono-chemical mechanisms [47], [29]. Previous studies have reported that high-frequency ultrasound can produce abundant radicals attacking specific chemical bonds, promoting oxidation [48], heterogeneous catalysis [26], and transesterification reactions [16]. However, as for heterogeneous deacetylation, the individual and synergistic contributions of ultrasound-driven thermal, chemical, and cavitation effects to the overall mechanism still need to be clarified and optimized [49].

**Table 2:** Recovery Yield, Deacetylation Efficiency and Molecular Weight of Chitosan Analogues of Conventional Extraction (CS-C), Low-Frequency Ultrasound Probe (CS-L), High-Frequency Ultrasound Plate (CS-H), and Ultrasound-Microwave Combination Reactor (CS-USMW) Compared with Commercial Chitosan (CS).

**Sample**	CS	CS-C	CS-USMW	CS-H	CS-L
**Degree of Deacetylation (%)**	≥ 75	77.97 ± 1.34^a^	73.25 ± 3.84^ab^	64.65 ± 0.51^bc^	24.80 ± 7.94^d^
**Recovery yield (%)**	−	53.40 ± 6.79^a^	31.10 ± 2.38^c^	35.40 ± 0.85^b^	15.20 ± 1.98^d^
**Molecular weight (kDa)**	385.32	115.82 ± 5.59^b^	101.61 ± 4.00^b^	139.17 ± 0.31^a^	−

The chitosan yield after deacetylation is shown in Table 2. The varying yields across different methods are primarily attributed to chitosan loss during the deacetylation process. On one side, during the washing step following alkali treatment, portions of chitosan are possibly soluble in water. Although chitosan tends to display higher solubility in acidic pH due to the protonation of amino groups [7], water-soluble chitosan has been extensively synthesized by deploying a range of reaction approaches, including depolymerization of the polymer chains [50], [51], [52]. Cavitation-induced shear and micro-jets physically break the β-1,4 glycosidic linkages, generating chitosan with low molecular weight, such as chitosan oligosaccharides (COS) that offers greater solubility in water [53], [54]. In this case, the cavitation induced by US-H, US-MW, and US-L has the effect of differently breaking the chitosan chain and generating COS compared to CVN, which will be further illustrated in Section 3.5. On the other side, fine chitosan particles suspended in the aqueous solution could also pass through the filtration membrane during centrifugation, leading to a reduction in overall yield. The particle size distribution of chitosan analogue extractions was further discussed in Section 3.3. Compared to CVN, all US-H, US-MW, and US-L resulted in lower chitosan yields, which is partly due to the intense shear forces created by high-speed microjets and shock waves during the collapse of cavitation bubbles breaking larger particles into smaller or even nanoscale particles [55]. Among the ultrasound treatments, the chitosan yield using US-H was relatively higher. This might be ascribed to the milder mechanical effects of high-frequency ultrasound being less destructive on chitin particles. Additionally, US-MW displayed higher recovery of chitosan compared to US-L (p < 0.05), because strong deacetylation exposes amino groups, which induce the aggregation under neutral conditions by electrostatic attraction and hydrogen bonding [56], [57].

In conclusion, effective deacetylation was obtained by CVN, US-H, and US-MW, and CS-C and CS-H demonstrated higher recovery yield compared to CS-USMW, achieving an effective chitin-to-chitosan conversion.

### 3.3 Particle size distribution

**Fig. 5:**
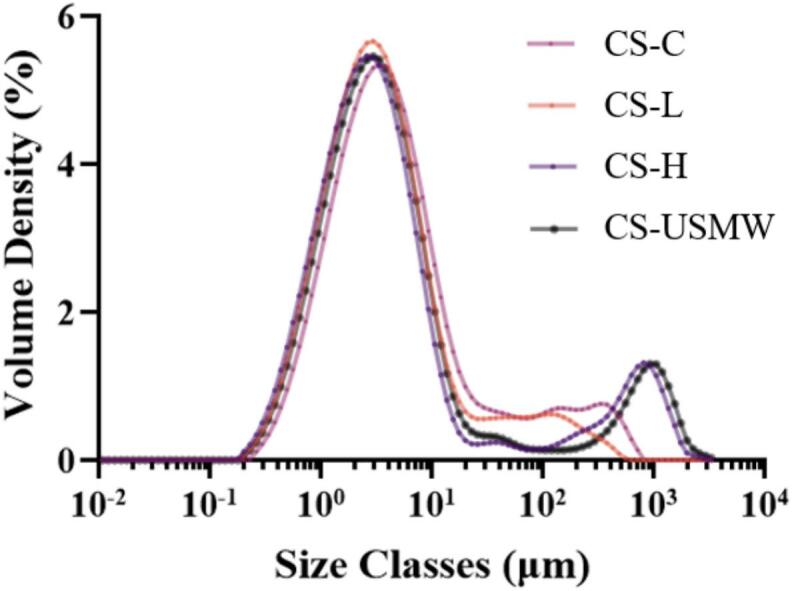
Particle size distribution of chitosan analogue extractions by conventional extraction (CS-C), low-frequency ultrasound probe (CS-L), high-frequency ultrasound plate (CS-H), and ultrasound-microwave combination reactor (CS-USMW).

To further verify the particle size reduction under different treatments contributing to the recovery loss, as Section 3.2, the particle size distribution of chitosan analogues is shown in Fig. 5. Across all conditions, micron-sized particles were predominantly obtained, exhibiting a peak with the largest area between 0.1–20 μm. Among ultrasound treatments, all the chitosan analogues’ particle size distribution showed a peak in the range of 2–3 μm, while the CS-C exhibited a distribution peak slightly shifted to a higher particle size range of 3–4 μm. In this study, ultrasound treatments only resulted in a slight reduction in the particle size of the obtained chitosan analogues, which provided part of evidence supporting the substantial loss of chitosan analogues as reported in Section 3.2. Nevertheless, numerous previous studies have demonstrated that ultrasound can effectively shear the chitosan particle to produce nanoscale chitosan by the mechanical shearing effect of ultrasound [55], [58], [30]. This discrepancy is possibly because the smaller chitosan particles generated during ultrasound treatment may have been lost during post-deacetylation washing, not retained for subsequent molecular weight determination to reflect their contribution in the analytical results.

It is noted that some peaks appeared at large particle size regions to form a wide size distribution. This is consistent with the known adhesive nature of chitosan particles, which tends to cause agglomeration in aqueous media [59]. Previous reports have demonstrated that particles with large surface area tend to form strong agglomerates during drying and display a poor redispersion capacity in aqueous solutions, due to extensive hydrogen bonding and van der Waals interactions [60], [61]. In our work, CS-H and CS-USMW chitosan analogues exhibited a broad size distribution with a secondary distribution observed in the range of 200–2000 μm. It is hypothesized that ultrasound cavitation destroyed the chitosan analogues crystallization (discussed in Section 3.4), exposing more amino groups, which increased the positive surface charge. This would disrupt the balance between attractive and repulsive forces by hydrogen bonding and electrostatic interactions, thereby promoting the coalescence of molecules into larger aggregates [55], [56]. In contrast, the CS-L sample appears to retain a higher proportion of acetylated groups, which limits the number of free amino groups available for electrostatic interactions. Therefore, CS-L concentrated on the smallest particle size classification and distribution under less charge-driven aggregation and more physical shearing of particles by US cavitation [30].

These findings demonstrate that both cavitation and DD play critical roles in determining the particle size distribution of chitosan powder. While ultrasound treatment effectively reduces particle size through shearing, it increases the likelihood of aggregation due to stronger intermolecular interactions.

### 3.4 Polymer crystallinity characterization

**Fig. 6:**
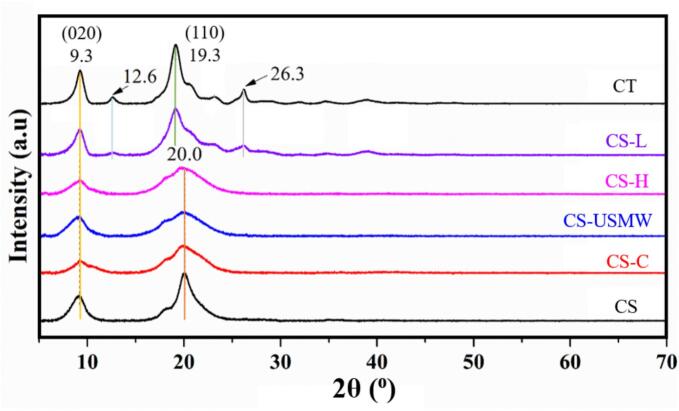
X-Ray diffraction (XRD) of commercial chitin (CT), commercial chitosan (CS), and chitosan analogue extractions by conventional extraction (CS-C), low-frequency ultrasound probe (CS-L), high-frequency ultrasound plate (CS-H), and ultrasound-microwave combination reactor (CS-USMW).

The complete XRD diffractogram for deacetylated analogues, as well as commercial chitosan and chitin, is shown in Fig. 6. The most prominent plane reflections for chitosan as previously reported are in the range 9.0° − 9.3° assigning to (020) crystallographic plane, and another reflection in 2θ range 19.0° − 20.3°, with less intensified reflections in the range 18.0° − 18.2°, assigning to (110) and (200) crystallographic plane respectively, which are characteristic peaks corresponding to chitosan [62], [63]. The percentage of crystallinity is determined by analysing the characteristic diffraction planes (020) and (110) in each sample diffractogram, shown in Table 3. Among the samples, commercial chitin (CT) exhibited the highest crystallinity (87.9%). This significant crystallinity is attributed to its highly ordered molecular structure, resulting from extensive intermolecular hydrogen bonding between acetamide groups within the polymer chains [64].

**Table 3:** Crystallinity Values of Commercial Chitin (CT), Commercial Chitosan (CS), and Chitosan Analogue Extractions by Conventional Extraction (CS-C), Low-Frequency Ultrasound probe (CS-L), High-Frequency Ultrasound Plate (CS-H) and Ultrasound-Microwave Combination Reactor (CS-USMW).

	**2θ angle (°)**		**Crystallinity (%)**
	**(020)**	**(110)**	
CT	9.2	19.2	87.9 ± 1.7^a^
CS-L	9.2	19.3	80.2 ± 1.2^b^
CS-H	9.2	20.2	75.1 ± 1.1^c^
CS-USMW	9.1	20.1	72.9 ± 1.5^c^
CS-C	9.2	20.3	77.2 ± 2.2^bc^
CS	9.1	20.0	81.2 ± 1.5^b^

In case of ultrasound treatments, CS-C, CS-USMW, and CS-H exhibited identical typical peak patterns to CS, demonstrating a similar pattern in the absence of any other crystallites and contaminants. However, CS-L showed different diffractogram patterns, with the absence of a peak detected around 18.0°, and other less intensified peaks were found majorly at 12.6°, 26.3°, and 38.8° in addition to chitosan characteristic peaks. This denotes the existence of chitin crystallite forms [65], [63]. Additionally, the peak intensities varied in strength depending on the treatment method used. For ultrasound-obtained chitosan analogues, the intensity of the diffraction peak decreased, indicating that the hydrogen bonding between molecules and the crystalline area was destroyed [66], namely, the degree of amorphization increased and the crystallinity decreased (shown in Table 3). The CS-C displayed a similar crystallinity to CS (*p* > 0.05), while CS-H and CS-USMW had relatively less crystallinity compared to CS (*p* < 0.05), and CS-USMW had the lowest crystallinity among all the treatments, but not significantly different compared to CS-H (*p* > 0.05). The reduced crystallinity of CS-H and CS-USMW might be because of the cavitation shearing force generated by ultrasound. According to the study of mechanical amorphization, the high-intensity grinding by prolonging time was employed, demonstrating that the crystallinity of chitosan was positively correlated with the mechanical strength [67]. It can be verified that the mechanical action by ultrasound cavitation could be the reason for the destruction of the crystalline area [68], [69]. In this case, mechanical shearing is more dominant in low-frequency US (US-MW) compared to high-frequency ultrasound (US-H), thus obtaining a lower crystallinity. Besides, it has been announced that microwave can also induce amorphization [70]. El Knidri et al. [37] have reported that a decreased 56.42% crystallinity by microwave irradiation was obtained compared to 64.91% crystallinity by conventional heating in chitosan recovery. Our result is different from the previous study, which reported that the peak intensity at (020) and (110) reflection decreased as DD increased [65], [71]. In those works, this was possibly attributed to the destruction of the polymer crystallinity area by further deacetylation.

In conclusion, the ultrasound cavitation can cause the chitosan polymer amorphization, showing a lower crystallinity, but with no significant difference compared to CS-C. However, the heterogeneous deacetylation may address the concerns about the inhomogeneity of extracted chitosan.

### 3.5 Molecular weight distribution

**Fig. 7:**
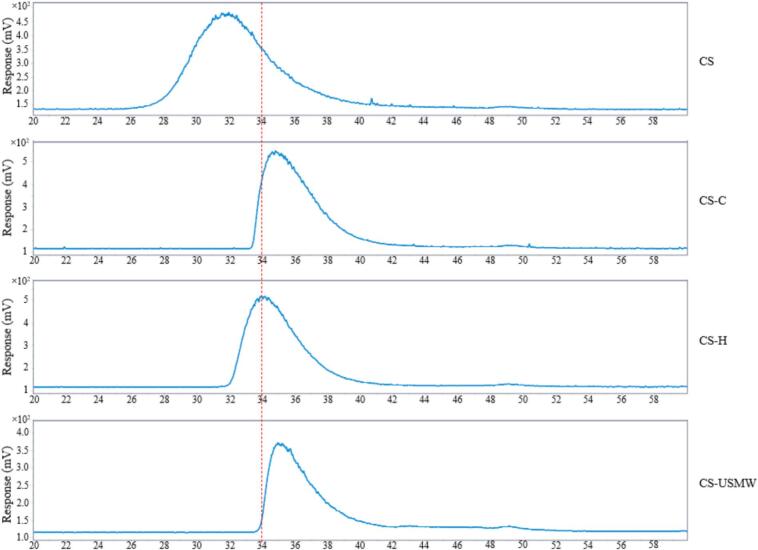
Molecular weight distribution of commercial chitosan (CS), and chitosan analogue extractions by conventional extraction (CS-C), high-frequency ultrasound plate (CS-H), and ultrasound-microwave combination reactor (CS-USMW).

The molecular weight distributions of CS, CS-S, CS-H, and CS-USMW, are shown in Fig. 7, and the molecular weight information was shown in Table 2. The chromatograms consistently exhibited bimodal distribution with a prominent peak appearing between 26 min and 42 min retention time, and a minor signal between 48 min and 50 min, indicating the presence of monomers. It is consistent with earlier reports of bimodality in chitosan prepared from crustacean shells [72].

CS exhibited a high molecular weight of 385.32 kDa, in agreement with the supplier's specification, supporting the validity of our method. By contrast, all extracted chitosan analogues (CS-C, CS-H, and CS-USMW) exhibited substantially lower molecular weights (<140 kDa), which were classified as medium-to-low molecular weight defined in previous reports [73]. This discrepancy may partly originate from the lower molecular weight of the starting commercial chitin used for conversion. Additionally, although many reports have widely demonstrated the effectiveness of ultrasound to degrade chitin and chitosan [74], [30], the substantial reduction of molecular weight observed is not solely attributable to ultrasound. In particular, CS-C also yielded chitosan with a greatly reduced molecular weight of 115.82 ± 5.59 kDa. Therefore, the strong alkali (50% concentrated NaOH) applied during deacetylation could also be another reason contributing to the reduction of the molecular weight of chitosan. This finding agrees with Tokatlı and Demirdöven [75] work, displaying similar degrees of deacetylation and molecular weights (127 kDa) for chitosan obtained by deacetylation with 50% sodium hydroxide at 100 °C for 720 min.

Among all treatments, CS-H maintained a relatively higher molecular weight of 139.17 ± 0.31 kDa among all the treatments (p < 0.05). Compared with CS-C, the reaction time was dramatically reduced from 3 h to 15 min, where a dynamically temperature-increasing system minimized the chitosan analogues exposure to the harsh hot-alkali environments, reducing the degradation of the polymer. Previous studies have confirmed that increasing deacetylation temperature generally contributes to a reduction in molecular weight [76], [77]. It is noted that high-frequency ultrasound has been reported to strongly depolymerize cellulose into glucose based on the wide generation of H· and OH· [78], as the sonochemistry is dominant at ultrasound frequencies above 100 kHz [29]. Different from cellulose depolymerization, Wu et al. [79] described that the degradation of chitosan by ultrasound is primarily driven by mechanical forces (shear, cavitation collapse, shock waves) rather than a radical oxidation process. And the cavitation induced by high-frequency ultrasound (more than 100 kHz) produces relatively mild mechanical shearing effects [80], [29], which might prevent severe cleavage of the chitosan backbone. Future studies should explore the mechanism of high-frequency ultrasound cavitation in deacetylation, which quantifies the contributions of chemical effect and mechanical shear induced by ultrasound. It can also be considered to optimise the alkali concentration, processing time, and high-frequency ultrasound power to achieve a milder deacetylation, producing medium to high molecular weight chitosan.

In contrast, the CS-USMW exhibited a lower molecular weight of 101.61 ± 4.00 kDa under comparable temperature and time conditions to CS-H (p < 0.05), with similar DD of chitosan analogues obtained. Previous studies have shown that rapid microwave heating can lead to inconsistent effects on chitosan molecular weight. For example, Lertjindaporn et al. [13] reported the cleavage of glycosidic bonds and a subsequent decrease in the polysaccharide's chain length after 8 min microwave radiation exposure (147.29 ± 14.64 kDa), while El Knidri et al. [37] documented a high molecular weight chitosan after 12 min microwave-assisted extraction. These findings suggest that the impact of microwave on chitosan polymers might strongly depend on the irradiation intensity employed. When microwave heating is combined with low-frequency ultrasound, the rapidly rising temperature is accompanied by intense cavitation. The strong mechanical shear generated by low-frequency ultrasound can accelerate chain scission, leading to pronounced degradation and the production of relatively low-molecular-weight chitosan. This interpretation is consistent with previous reports of ultrasound-induced depolymerization [81], [79].

As for CS-L, it is challenging to obtain molecular weight information by chromatography because a large number of acetyl groups remained in the extraction as discussed in Section 3.2, resulting in tricky solubility. Based on previous molecular weight determination based on intrinsic viscosity methods [18], [21], [22], it is suggested that low-frequency ultrasound is more effective in depolymerization than in deacetylation. Therefore, further work can be explored by combining ultrasound and microwave to leverage the heating capability of microwave and the depolymerization ability of ultrasound on the chitosan backbone to produce bio-active low molecular weight chitosan in a short time.

### 3.6 Thermal properties analysis

**Fig. 8:**
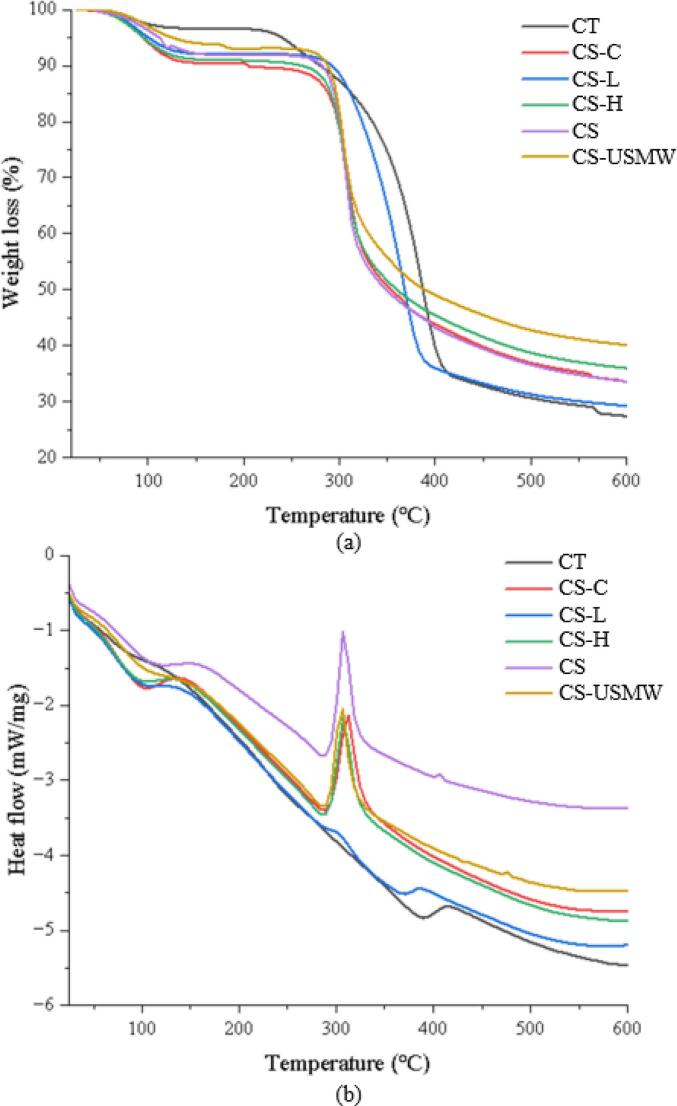
TGA-DSC curves of commercial chitin (CT), commercial chitosan (CS), and chitosan analogue extractions by conventional extraction (CS-C), low-frequency ultrasound probe (CS-L), high-frequency ultrasound plate (CS-H), and ultrasound-microwave combination reactor (CS-USMW). (a) displays TGA curves of chitosan analogues under ultrasound treatments; (b) displays DSC curves of chitosan analogues under ultrasound treatments.

The TGA-DSC images of the deacetylated analogues subjected to ultrasound treatments are presented in Fig. 8. The first thermal and mass loss event registered in all samples occurred between 65–130 °C, which corresponds to the dehydration of water molecules within the samples. Polysaccharides generally have a strong affinity for water, and these macromolecules may have a disordered structure in the solid state, making them easily hydrated [82], [83]. Notably, in the DSC image, a larger endothermic peak area and higher endothermic peak temperature in CS suggest the stronger water-holding capacity and water-polymer interaction compared to ultrasound-obtained analogues. This can be attributed to a larger molecular weight [84], which also corresponds to the results in Section 3.5.

The second mass loss event primarily corresponded to the decomposition temperature of chitosan, reflecting the thermal stability of the obtained analogues. The mass loss of CS, CS-C, CS-H, and CS-USMW consistently occurred at 290.72 °C, 288.67 °C, 291.63 °C, and 288.68 °C, associated with the decomposition of amino residues in the chitosan chains. It aligns with the degradation temperature of chitosan in existing studies [85], [86]. In contrast, CS-L chitosan exhibited a higher and wider mass loss temperature range between 316.86 °C − 374.75 °C. The curve of CS-L is consistent with TGA curve of commercial chitin (CT), as well as previous study reporting chitin extracted by traditional chemical extraction and microwave-assisted extraction [13], which are linked to the decomposition of N-acetyl residues according to previous studies [83]. Since amino residues are less thermally stable than N-acetyl residues, the decomposition of N-acetyl groups occurs at higher temperatures [84].

Analysing the DSC curves further reveals differences in the peak area and peak temperature of the endothermic events within this range, which reflect changes in thermal stability due to variations in acetyl content and degree of polymerization. At around 290 °C, indicating the amino group degradation, CS exhibited the largest peak area with the highest heat release during decomposition (ΔH_CS_ 158.23 J/g at 294.86 °C) compared to other chitosan analogues (ΔH_CS-C_ 147.88 J/g at 294.70 °C, ΔH_CS-H_ 147.37 J/g at 293.32 °C, ΔH_CS-USMW_ 137.52J/g at 292.54 °C). It is potentially due to its greater degree of polymerization compared to other chitosan analogues [87]. Additionally, the exothermic peak position of CS-USMW shifted slightly toward lower temperatures and presented a reduced peak area, indicating relatively limited thermal stability, which suggests that the synergistic effects of the low-frequency ultrasound shearing action and the microwave-induced thermal effect produce analogues with reduced thermal stability. Notably, CS-L displayed a significantly smaller peak area at lower temperature (ΔH_CS-L_ 66.13 J/g at 289.87 °C), with another weak peak at approximately 380 °C corresponding to decomposition of N-acetyl groups. This two-small-peaks curve was reported in Guinesi and Cavalheiro [88] work, indicating a linear relationship between peak area and height with the DA. This is possibly because low polymerized molecules with inhomogeneous (low crystallinity) and incomplete deacetylation by low-frequency ultrasound undergo multi-step decomposition of amino and N-acetyl groups within a highly overlapping temperature range, and the thermal effect diffuses to the baseline to form a relatively flat peak. The thermal degradation is largely affected by the crystallinity, morphology, and molecular weight [89].

In summary, the TGA-DSC curves of CS-C, CS-H, and CS-USMW basically resembled those of CS, while CS-L exhibited incomplete deacetylation displaying similar curves to CT. CS demonstrated superior thermal stability compared to other obtained analogues, attributed to its higher degree of polymerization, while the CS-USMW structure slightly tended to be thermally unstable.

### 3.7 Surface morphology evaluation

**Fig. 9:**
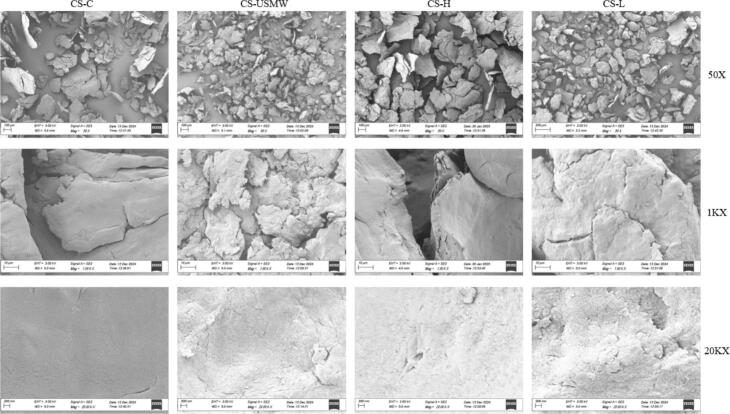
Surface morphology of chitosan analogue extractions by conventional extraction (CS-C), low-frequency ultrasound probe (CS-L), high-frequency ultrasound plate (CS-H), and ultrasound-microwave combination reactor (CS-USMW), at different magnifications (The treatments are labelled above the columns, and the magnifications are labelled at the end of each row).

The surface morphology of chitosan analogues at different magnifications (50X, 1KX, and 20KX) was analysed using SEM and represented in Fig. 9. At lower magnification (50X), the CS-C exhibited loosely distributed, irregularly shaped, and relatively large particles, which basically aligns with previously reported non-porous flakes micrographs [90], [37], [91]. The heating reflux showed a limited effect to expand and disaggregate particles. Furthermore, at an intermediate magnification (1KX), the CS-C sample displayed smooth-edged lamellar structures, which is a typical flake morphology of chitosan. In addition, a dense and smooth surface was observed in the CS-C sample at higher magnification (20KX) compared to ultrasound treated objectives, which suggests that gradual thermal alkali diffusion over 3 h induced slower surface swelling and fewer visible defects.

In contrast, both CS-USMW and CS-L samples showed a significant reduction in particle size under low-frequency ultrasound shearing. CS-L displayed more uniform dispersion due to ultrasound shear forces, which aligns with previous evidence that ultrasound treatment can shear chitosan analogues particles with narrow and smaller size distributions. It showed a similar structure to previous macroscopic chitosan morphology reported after sonication treatment [92]. CS-USMW showed some degree of aggregation because of strong deacetylation by heating. The CS-H sample at lower magnification had larger particle sizes with aggregation, further proving that the US-H primarily provides heating and homogenization with limited fragmentation effects, which is consistent with previous observations. Furthermore, both CS-L and CS-H samples showed rough stacking with minor cracks, indicating the slight morphological modifications under cavitation. According to Vallejo-Domínguez et al. [30], the surface of chitosan was eroded with obvious porosity and prolonged ultrasound treatment. It was explained that the cutting force, shock wave, and turbulence strengthen the alkali corrosion. The CS-USMW sample exhibited extreme irregular edges and fragmented internal structures, reflecting the combined effects of rapid thermal expansion and ultrasound shear forces, causing significant disruption of chitosan's microstructure. However, previous studies showed similar morphology between conventional heating and microwave radiation assisting deacetylation [93], [37]. The severe structural damage may be attributed to the synergistic effect of the microwave and ultrasound combination. In comparison to CS-H showing few surface cracks and voids, CS-USMW and CS-L samples exhibited rough surfaces with prominent cracks and pores. It is likely due to low-frequency ultrasound cavitation and bubble collapse causing stronger mechanical disruption on the chitosan structure, while the high-frequency ultrasound generates more uniform cavitation, beneficial to the interface reaction. In overall, the surfaces of the ultrasound treated chitosan analogues exhibit signs of swelling, damage, and delamination. It is possibly because the rapid heating within 15 min may have caused internal expansion, accompanied by varying degrees of cavitation, leading to the formation of microcracks and increased surface exposure [43], [30]. And it also displayed a visual reflection on increased accessibility and porosity under ultrasound to promote the proceeding of the deacetylation reaction.

## 4 Conclusions

This study systematically evaluated the heterogeneous deacetylation of chitin using various ultrasound-assisted systems, including a low-frequency ultrasound probe (US-L), high-frequency ultrasound plate (US-H), and ultrasound-microwave combination reactor (US-MW), compared to conventional alkaline heating (CVN). Both US-MW and US-H achieved effective deacetylation within just 15 min, reaching degrees of deacetylation (DD) of 73.25% and 64.65%, respectively. In contrast, US-L was not effective for producing chitosan in a short time due to limited thermal effect and cavitation intensity. Further structural and thermal stability analyses by FTIR, XRD, HP-SEC, and TGA-DSC confirmed that US-H, US-MW, and CVN generated comparable medium-to-low molecular weight chitosan with a more amorphous structure and less thermal stability compared to commercial chitosan (CS), while US-L retained characteristics of chitin. Among the chitosan analogues produced by ultrasound treatments, US-H potentially preserves the chitosan polymer and morphology during deacetylation, compared to surface erosion and fragmentation of US-MW combined thermal and mechanical effects. Multidimensional recovery and structural characterisation suggest that US-H presents a rapid and relatively mild deacetylation technique, enabling promising deacetylation without extensive polymer breakdown for further process optimization. Conversely, US-MW exhibited pronounced fragmentation, supporting its application for controlled production of low-molecular-weight chitosan. This work provides a fundamental strategy in developing efficient chitosan production, and further research can be conducted in technology optimization, chemical recycling and mechanism exploration to achieve precise control over the degree of deacetylation. Also, energy analysis and techno-economic assessment can be integrated into environmental or economic benefits at scale.

## CRediT authorship contribution statement

**Wenrui Dong:** Conceptualization, Investigation, Writing – original draft. **Eugenia Mazzara:** Investigation, Writing – review & editing. **Alejandro Sánchez-Baca:** Investigation. **Kona Mondal:** Investigation. **Mar Villamiel:** Supervision, Funding acquisition. **Ramesh Babu:** Supervision, Funding acquisition. **Da-Wen Sun:** Writing – review & editing, Supervision, Funding acquisition. **Brijesh K. Tiwari:** Supervision, Funding acquisition, Resources, Writing – review & editing.

## Declaration of competing interest

The authors declare that they have no known competing financial interests or personal relationships that could have appeared to influence the work reported in this paper.
